# Alpha-mangostin from mangosteen (*Garcinia mangostana Linn.)* pericarp extract reduces high fat-diet induced hepatic steatosis in rats by regulating mitochondria function and apoptosis

**DOI:** 10.1186/s12986-016-0148-0

**Published:** 2016-12-01

**Authors:** Shin-Yu Tsai, Pei-Chin Chung, Eddy E. Owaga, I-Jong Tsai, Pei-Yuan Wang, Jeng-I Tsai, Tien-Shun Yeh, Rong-Hong Hsieh

**Affiliations:** 1 School of Nutrition and Health Sciences, Taipei Medical University, 250 Wu-Hsing Street, Taipei, 110 Taiwan; 2 Institute of Food Bioresources and Technology, Dedan Kimathi University of Technology, P.O. Box 657-10100 Nyeri, Kenya; 3 Yuan Lyu Technology Corporation, 10F-3, 120 Chung Cheng 1st Road, Kaohsiung, 802 Taiwan; 4 Institute of Anatomy and Cell Biology, School of Medicine, National Yang-Ming University, Taipei, 112 Taiwan

**Keywords:** α-mangostin, Fat infiltration, Mitochondria dysfunction, Mitochondrial pathway apoptosis, Antioxidant enzymes

## Abstract

**Background:**

Non-alcoholic fatty liver disease (NAFLD) is caused by multiple factors including hepatic oxidative stress, lipotoxicity, and mitochondrial dysfunction. Obesity is among the risk factors for NAFLD alongside type 2 diabetes mellitus and hyperlipidemia. α- mangostin (α-MG) extracts from the pericarps of mangosteen (*Garcinia mangostana Linn.)* may regulate high fat diet-induced hepatic steatosis; however the underlying mechanisms remain unknown. The aim of this study was to investigate the regulatory effect of α-MG on high fat diet-induced hepatic steatosis and the underlying mechanisms related to mitochondrial functionality and apoptosis in vivo and in vitro.

**Methods:**

Sprague Dawley (SD) rats were fed on either AIM 93-M control diet, a high-fat diet (HFD), or high-fat diet supplemented with 25 mg/day mangosteen pericarp extract (MGE) for 11 weeks. Thereafter, the following were determined: body weight change, plasma free fatty acids, liver triglyceride content, antioxidant enzymes (superoxide dismutase, SOD; glutathione, GSH; glutathione peroxidase, GPx; glutathione reductase GRd; catalase, CAT) and mitochondrial complex enzyme activities. In the in vitro study, primary liver cells were treated with 1 mM free fatty acid (FFA) (palmitate: oleate acid = 2:0.25) to induce steatosis. Thereafter, the effects of α-MG (10 μM, 20 μM, 30 μM) on total and mitochondria ROS (tROS, mitoROS), mitochondria bioenergetic functions, and mitochondrial pathway of apoptosis were examined in the FFA-treated primary liver cells.

**Results:**

The MGE group showed significantly decreased plasma free fatty acids and hepatic triglycerides (TG) and thiorbarbituric acid reactive substances (TBARS) levels; increased activities of antioxidant enzymes (SOD, GSH, GPx, GRd, CAT); and enhanced NADH-cytochrome *c* reductase (NCCR) and succinate-cytochrome *c* reductase (SCCR) activities in the liver tissue compared with HFD group. In the in vitro study, α-MG significantly increased mitochondrial membrane potential, enhanced cellular oxygen consumption rate (OCR), decreased tROS (total ROS) and mitoROS (mitochondrial ROS) levels ; reduced Ca^2+^ and cytochrome *c* (cyt *c*) release from mitochondria, and reduced caspases 9 and 3 activities compared with control group.

**Conclusion:**

These findings demonstrate α-MG attenuated hepatic steatosis in high fat-diet fed rats potentially through enhanced cellular antioxidant capacity and improved mitochondrial functions as well as suppressed apoptosis of hepatocytes. The findings of study represent a novel nutritional approach on the use of α-MG in the prevention and management of NAFLD.

## Background

Non-alcoholic fatty liver disease (NAFLD) is among the most common chronic liver diseases and its prevalence is associated with the increase in obesity and other metabolic syndrome conditions [1, 2] The hallmark of NAFLD is hepatic steatosis, which manifests as excessive triglyceride accumulation in the hepatocytes [2]. The pathogenesis of NAFLD is generally described by ‘two-hit’ theory represented by initial step of hepatic lipid accumulation and thereafter hepatic cellular injury due to lipotoxicity mediated by lipid peroxidation and pro-inflammatory factors [1]. Hepatic steatosis mainly results from disrupted lipid metabolism in the hepatocytes leading to imbalance between intrahepatic triglyceride (TG) accumulation and clearance [3]. Among the underlying reasons for the fat accumulation is the failure of long chain fatty acids catabolism due to impaired hepatic mitochondrial β-oxidation [1]. The increased FFA flux in the hepatocytes increase the production of free radicals and other reactive oxygen species (ROS) leading to intracellular redox imbalance. Due to the reactive nature of the ROS, the antioxidant enzyme activities are suppressed resulting in oxidative stress and hepatic cellular injury [4]*.*

Hepatic lipid accumulation in NAFLD setting has been associated with mitochondrial dysfunction arising mainly from cellular oxidative damage [1]. Mitochondria are key sources of ROS in the cell mainly due to mitochondrial uncoupling disturbances and inhibition of the respiratory chain leading to free radical leakage in the electron transport chain [5]. A previous study found rat fed on high fat diet had increased levels of free fatty acid, oxidative stress, and decreased antioxidant enzymes activity, which collectively induced mitochondrial proton leakage [6]. The imbalance between the mitochondria antioxidant capacity and generated ROS affects the mitochondria integrity and catabolic functions such as β-oxidation of fatty acids thereby resulting in excessive hepatic triglyceride accumulation [4].

Apoptosis of hepatocytes is a critical feature of NAFLD and is associated with structural changes in the liver tissues leading to physiological and pathological conditions [7]. The role of mitochondria-dependent apoptosis in the development of hepatic steatosis has been hypothesized by several authors [8]. Oxidative stress can interfere with mitochondria membrane integrity including loss of mitochondrial membrane potential (Δψm), release of Ca^2+^, activation of caspases 9 and 3; reduction of Bcl-2/Bax ratio and release of cytochrome c leading to programmed hepatocytes cell death [9]. The main consequence of hepatocyte apoptosis is dysregulated lipid clearance resulting in triglyceride accumulation. On the other hand, oxidative stress can also cause hepatocytes apoptosis through the redox signalling [8].

In recent years, several studies have explored the modification of the risk factors such as oxidative stress as potential therapeutic targets for NAFLD through the use of antioxidants and dietary bioactive compounds [1, 4]. Mangosteen (*Garcinia mangostana Linn*.) is a tropical fruit that contains a wide range of bioactive compounds such as xanthones, anthocyanins, tannins and the beneficial health effects have been linked to the anti-oxidative, anti-bacterial, anti-cancer and anti-adipogenic effects [4, 10]. α- mangostin (α-MG) is the main constituent of the fruit hull of the mangosteen, and possesses a wide variety of pharmacological effects including inhibition of inflammation; inhibition of ROS through enhanced anti-oxidative enzymes activities [11]; and improvement of metabolic disorders of high fat-diet fed mice [4]. Previous studies showed α-MG significantly reduced lipid peroxidation and improved antioxidant capacity in fat-infiltrated liver [4, 12]. Choi et al. found α-MG treatment could alleviate obesity in a mice model through induction of adenosine 5’-monophosphate (AMP)-activated protein kinase (AMPK) protein [12]. Nevertheless, the effect of mangosteen on hepatic lipid homeostasis in NAFLD setting has not been elucidated and the underlying mechanisms related to mitochondria functionality and apoptosis remain unknown. Thus, our study aimed at investigating the regulatory effects of mangosteen pericarp extract on hepatic fat- accumulation in high-fat diet fed rats, and further examine the underlying mechanisms related to mitochondrial functionality and apoptosis in vivo and in vitro.

## Methods

### Reagents

Palmitic acid, oleic acid, pure α-MG (>99%), 1% Penicillin/Streptomycin (PS), and dimethyl sulfoxide (DMSO) were obtained from Sigma-Aldrich (US). Leibovitz’s L-15 medium was purchased from ThermoFisher, (US), whereas fetal bovine serum (FBS) was obtained from GIBCO Co. (US). Mangosteen pericarp extract was obtained from Shinn Nan World Trade Co., Ltd. (Taipei, Taiwan) and contained 84% total mangosteen xanthones as α-mangostin.

### Animal experiment

The animal experiment protocol was reviewed and approved by the Institutional Animal Care and Use committee (IACUC/IACUP) at Taipei Medical University (IACUC approval No: LAC-2015-0109). Adult male SD rats (240.2 ± 1.5 g, 6–7 weeks old were obtained from BioLASCO Taiwan Co., Ltd. (Taiwan) and maintained under specific-free conditions at 22 °C on a 12 h light–dark cycle with *ad libitum* access to food and water. After 1 week of acclimatization, the rats were equally divided into three groups (*n* = 8): (i)AIN 93 M diet (control) from MP Biomedicals (US) (ii) High fat diet group (HFD) - AIN93M modified high fat diet (iii) MG group (MGE) -25 mg/day mangosteen extract with AIN93M modified high fat diet. The caloric distribution of the control group was 77%: 9%:14% of energy provided as carbohydrate: fat: protein, respectively whereas the caloric distribution of the HFD group was 28%: 60%:11%, of energy provided as carbohydrate: fat: protein, respectively. Lard was used as the major fat source in the diet. The amount of food consumed by the rats in the respective groups were not significantly different i.e. control group - 19.79 ± 0.34 g/per day; HFD group- 19.67 ± 0.54 g/per day; and MGE group- 19.41 ± 1.02 g/per day. The rats were weighed every week during the experiment period. After 11 weeks of feeding, the rats were fasted overnight and sacrificed. The liver tissues were removed, weighed and thereafter subjected to histology examination. All samples were stored at −80 °C prior to analysis of hepatic triglyceride and free fatty acids content, hepatic oxidative damage expressed as Thiobarbituric acid-reactive substances (TBARS), antioxidant enzyme activities, and mitochondrial complex enzyme activities.

### Isolation of primary hepatocytes and Free fatty acid (FFA)-treatment experimental design

Primary hepatocytes were isolated from 6 to 8 weeks old male SD rats weighing 150–200 g, through portal vein collagenase perfusion of liver as per the method of Seglen [13]. Hepatocytes were cultured for 24 h in Leibovitz’s L-15 medium supplemented with 1 μmol/L dexamethasone and 2.5% FBS (supplemented with 5.6 mM/L galactose, and 1% penicillin/streptomycin). A typical yield was about 4–5 × 10^7^ hepatocytes/rats with >80% cell viability as determined by trypan blue exclusion assay. In order to induce fat infiltration to simulate hepatic steatosis, rat primary hepatocytes were placed in 6-well plates (2 × 10^5^ cells) and cultured for 24 h in 10% FBS media supplemented with 1 mM free fatty acid (FFA) (palmitate: oleate acid =2:1) as previously described [14]. The primary hepatocytes were treated with different α-MG concentrations (10 μM, 20 μM and 30 μM) were dissolved before use DMSO for 24 h. These dosages fall within the ranges previously studied by other authors [15]. Analyses were conducted for cellular oxygen consumption rate (OCR), total and mitochondrial ROS, cytochrome c release, caspase-3 and −9, and mitochondrial membrane potential.

### Analysis of hepatic triglyceride and plasma free fatty acids profile

The plasma free fatty acid concentrations were determined by enzymatic colorimetric methods using commercial kits (Randox, UK). The lipids were extracted from liver samples using chloroform–methanol method [16]. The liver TG levels in the lipid extracts were determined by enzymatic colorimetric methods using commercial kits (Randox, UK).

### Hepatic histology

Liver tissues were fixed overnight at room temperature in 10% formaldehyde and embedded in paraffin. Thick sections (8 μm) were stained with hematoxylin & eosin and mounted on glass slides and examined using a microscope (Leica ICC50 HD, Germany) (original magnification × 200).

### Hepatic TBARS assay

As a marker of oxidative damage from lipid peroxidation, the TBARS concentrations were measured in the liver tissue and primary hepatocytes using the method of Park et al. [17]. First, the supernatant (20 μL) was mixed with 0.22% H_2_SO_4 (_800 μL), phosphotungstic acid (PTA) (100 μL) and thiobarbituric acid (TBA) (200 μL). The reaction mixture was heated at 95 °C for 1 h, and cooled for 10 min. Thereafter butanol (400 μL) was added to the mixture and vortexed. The mixture was centrifuged at 25 °C for 15 min (650 × g) and the supernatant fluorescence measured using TBARS Assay Kit (Cayman, US) at excitation and emission wavelengths of 530 nm and 590 nm, respectively.

### Assay of hepatic antioxidant enzymes activities

Liver tissue (0.5 g) was homogenized in 1.5 mL buffer (20 M Tris-base,7 VM NaCl, 1% Triton X-100, pH 7.2, 0.1% protease inhibitor) and incubated at 4 °C, vortexing every 10 min up to 90 min. The homogenate was centrifuged at 4 °C, for 15 min (3000 × g). The supernatants were collected and stored at −30 °C for further analysis. The amount of protein in supernatant was measured using bicinchoninic acid (BCA) kit (ThermoFisher, US). Superoxide dismutase (SOD) activity was analyzed using commercial kit (ENZO, UK). The glutathione (GSH) and catalase (CAT) activities were measured using respective commercial kits (Cayman, US). The glutathione reductase (GRd) and glutathione peroxidase (GPx) activities were determined using respective commercial kits (Randox, UK).

### Analysis of hepatic mitochondrial complex enzyme activity

In the determination of nicotinamide adenine dinucleotide-cytochrome c reductase (NCCR) activity, 900 μL test solution (1 mM NADH, 1.5 mM potassium cyanide, 50 mM potassium phosphate buffer, pH 7.4) was added to 200 μg of liver mitochondrion extract and incubated at 37 °C for 3 min. Thereafter, 1 mM oxidized cytochrome *c* (100 μL) was added. The activity absorbance was measured spectrophotometrically at 550 nm for 3 min. In the succinate-cytochrome c reductase (SCCR) activity assay, 900 μL test solution (22 mM succinate, 1.66 mM potassium cyanide, 44 mM potassium phosphate buffer, pH 7.4), was mixed with liver mitochondrion extract (200 μg) and 0.5 mM oxidized cytochrome c (100 μL), and incubated at 37 °C for 2 min. The SCCR activity was measured spectrophotometrically at 550 nm for 3 min.

### Determination of cellular oxygen consumption rate (OCR)

The FFA-treated rat primary hepatocyte (5 × 10^4^ cells per well) were cultured in plates for the analysis of the cellular oxygen consumption rate using XFe24 Analyzer plates (Seahorse Bioscience, US). The analyzer was sequentially injected with the three mitochondrial inhibitor compounds (oligomycin, carbonylcyanide p-trifluoromethoxyphenylhydrazone, CCCP; and antimycin A) through the assigned ports. The OCR was expressed as pMole/min.

### Total ROS (tROS) and mitochondrial ROS (mitoROS) assay

tROS was determined in the FFA-treated primary hepatocytes by analysis of 2,7-dichlorodihydrofluorescein diacetate (H_2_DCFDA) using a commercial kit (Cayman, US). mitoROS was examined using MitoSOX™ Red commercial kit (Cayman, US)..

### Assay of mitochondrial membrane potential, cytochrome c (cyt *c*), and caspase (3 and 9) activities

Mitochondrial membrane potential was determined using MitoProbe™ JC-1 assay kit. (ThermoFisher, US). Cyt *c* assay was evaluated using cyt *c* releasing apoptosis assay kit (Biovision, US). The cyt *c* release was determined by western blotting of the cell homogenate of the cytosolic and mitochondrial fractions. Caspases-3 and 9 assays were evaluated using caspase-3/CPP32 and 9 colorimetric assay kits, respectively (Biovision, US). The cells were sonicated to obtain protein concentration of 100 μg/mL. After addition of reaction buffer and DTT to the protein extract, DEVD-pNA (caspase 3) or LEHD-pNA (caspase 9) substrate were added to the mixture and incubated at 37 °C for 2 h. The samples absorbance were read at 405 nm using a spectrophotometer.

### Statistical analysis

Data are expressed as the mean ± standard deviation. The differences between the mean values were evaluated by ANOVA followed by Duncan’s multiple range test. In all analyses, *p*-values < 0.05 were considered statistically significant.

## Results

### Mangosteen pericarps extract markedly reduced body weight gain, plasma free fatty acids (FFA) levels, hepatic triglyceride (TG) accumulation

**Fig 1:**
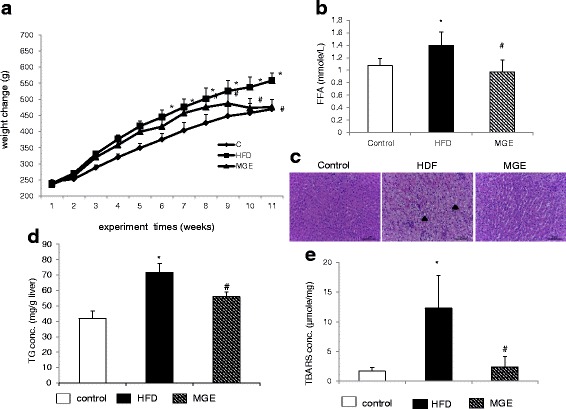
Effect of mangosteen pericarp extracts on: **a** body weight change, **b** FFA in plasma, **c** representative images of hematoxylin and eosin stained liver tissue. Original magnification, ×200, **d** Liver TG concentration, **e** Liver TBARS concentration. FFA: free fatty acid, TG: triacylglycerol, TBARS: thiobarbituric acid reactive substances. **c**: control; HFD: high fat diet; MGE: HFD supplemented with 25 mg/day mangosteen pericarp extract. Values are mean ± SD (*n* = 8); (*P < 0.05 versus control ; ^#^ *P* < 0.05 versus HFD)

Mangosteen pericarps extract supplementation significantly decreased body weight in the MGE group compared to HFD group (Fig. 1a). After feeding the SD rats on high fat diet (HFD) for 11 weeks, plasma FFA (Fig. 1b) and hepatic TG concentration (Fig. 1d) were markedly increased in HFD group thereby confirming the primary goal of inducing fat accumulation in the animal model was successful. This is further supported by the hepatic histology images that showed increased lipid deposition in the hepatocytes of the HFD group showed compared to control and MGE groups (Fig. 1c). Mangosteen pericarps extract supplementation significantly reduced hepatic TG concentration (Fig. 1d) and plasma FFA levels (Fig. 1b) MGE group compared to HFD group. Mangosteen pericarps extract also significantly reduced TBARS levels, a marker of lipid peroxidation, in the MGE group (Fig. 1e).

### Mangosteen pericarps extract increased hepatic antioxidant enzyme activities and reduced ROS in rat liver tissue

**Fig 2:**
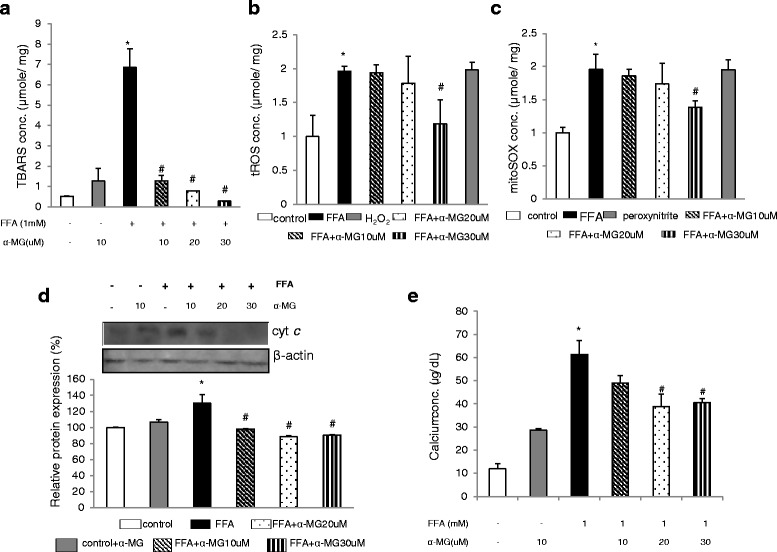
α-MG treatment decreased **a** TBARS, **b** tROS, **c** mitoROS, **d** cyt c, **e** Ca^2+^ in FFA-treated primary hepatocyte. The primary liver cells were treated with α-MG (10, 20, 30 μM) for 24 h. Thiobarbituric acid reactive substances (TBARS), tROS and mitoROS were quantified by flow cytometry, whereas Ca2+ concentration were quantified by colorimetry assays. Cyt c level was quantified by western blotting. Values are means ± SD, *n* = 3. (*P < 0.05 versus primary liver cells ; ^#^ *P* < 0.05 versus FFA-induced hepatocyte)

Mangosteen pericarps extract treatments increased antioxidant enzymes (SOD, GSH, GPx, GRd, CAT) activities in the rat liver tissue in MGE group compared to HFD group (Table 1). These results demonstrate mangosteen extract could confer a protective effect by enhancing hepatic antioxidant enzymes activities. In the in vitro study, α-MG treatment decreased TBARS concentration in FFA-treated hepatocytes (Fig. 2a) and decreased tROS (Fig. 2b) and mitoROS (Fig. 2c) concentration in FFA -treated hepatocytes. Overall, our study found α-MG decreased oxidative stress in FFA -treated hepatocytes.

**Table 1:** Effect of mangosteen peel extract on hepatic antioxidant enzyme activity in high fat diet-fed rats

	Control	HFD	MGE
GSH (mole/mg protein)	96.9 ± 2.2	93.0 ± 0.7*	96.5 ± 1.6^#^
GPx (U/mg protein)	0.98 ± 0.08	0.85 ± 0.14*	0.95 ± 0.08^#^
GRd (U/g protein)	0.82 ± 0.2	0.66 ± 0.14*	0.96 ± 0.21^#^
SOD (Unit/min/mg protein)	0.02 ± 0.03	0.14 ± 0.05*	0.20 ± 0.03^#^
CAT (Kunits/mg protein)	19.5 ± 2.8	14.3 ± 5.3*	19.9 ± 2.6^#^

Values are mean ± SD (*n* = 8); means without a common letter differ (*p* < 0.05). GSH: glutathione; GPx: glutathione peroxidase; GRd: glutathione reductase; SOD: superoxide dismutase; CAT: catalase. HFD: high fat diet; MGE: HFD supplemented 25 mg/day mangosteen extract diet (*P < 0.05 versus control ; ^#^ *P* < 0.05 versus HFD)

### α-MG suppressed mitochondrial pathway of apoptosis in FFA-treated hepatocytes

**Fig 3:**
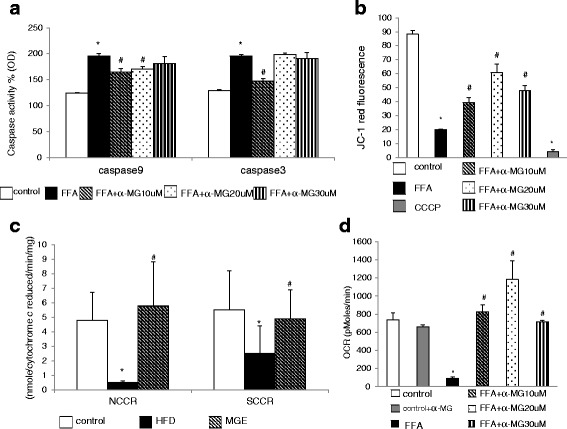
Effect of α-MG treatment on : **a** caspases 9 and 3 activities, **b** mitochondria membrane expressed as JC-1 fluorescence, **d** OXPHOS expressed as oxygen consumption rate in FFA-treated hepatocytes. The primary liver cells were treated with α-MG (10, 20, 30 μM) for 24 h. OXPHOS was quantified by seahorse XFe24 Analyzer. Caspases 9 and caspases 3 were quantified by Elisa assays. (*P < 0.05 versus primary liver cells ; ^#^ *P* < 0.05 versus FFA-treated hepatocytes). Values are means ± SD, *n* = 3. Effect of mangosteen pericarp extract on mitochondrion complex enzymes (NCCR, SCCR) **c**. OXPHOS: oxidative phosphorylation. NCCR: NADH-cytochrome *c* reductase. SCCR: succinate-cytochrome *c* reductase. Values are mean ± SD (*n* = 8); (*P < 0.05 versus control; ^#^ *P* < 0.05 versus HFD)

α-MG treatments (10 μM, 20 μM and 30 μM) decreased Ca^2+^ (Fig. 2e) and cytochrome c levels (Fig. 2d), decreased caspases 9 and 3 activities (Fig.3a), and decreased mitochondrial membrane potential (Fig. 3b) in FFA-treated primary hepatocytes. Our results implied α-MG suppressed the early phase of mitochondrial-dependent apoptosis.

### α-MG treatment enhanced mitochondrial function and enzyme activities

α-MG treatment increased NCCR (mitochondrial complex I and III) and SCCR (mitochondrial complex II and III) activities in MGE group compared to HFD group (Fig. 3c). Our results showed significantly increased mitochondrial oxygen consumption rate in the 20 μM α-MG treatment compared to control in FFA-treated hepatocytes (Fig. 3d). These data show α-MG of mangosteen pericarps extract treatment could improve efficiency of mitochondrial functions such as oxidative phosphorylation and enzyme activities.

## Discussion

We evaluated the regulatory effects of mangosteen pericarp extract on hepatic fat-accumulation in high-fat diet fed rats, and further examine the underlying mechanisms related to mitochondrial functionality and apoptosis in vivo and in vitro. In this study, the weight change in the MGE group was markedly lower than in the HFD-control diet (Fig. 1a), and MGE further suppressed accumulation of free fatty acids (Fig. 1b), lipid deposition in hepatocytes (Fig. 1c) and triglyceride (Fig. 1d) compared to the levels in the HFD group. We report here for the first time, that the MGE suppressed effect on hepatic triglyceride accumulation could be directly or indirectly attributed to modulation of the antioxidant capacity, mitochondrial bioenergetic functionality and mitochondria-dependent apoptosis. Our findings on suppressed triglyceride were in agreement with a previous study that showed improved biochemical serum profiles (total cholesterol, triglyceride, and fatty acid) in high fat diet fed mice after supplementation with α-MG (50 mg/kg) for 80 days [4]. Choi et al. also reported anti-obesity effects of α-MG in HFD induced obese mice by activating SirT1-AMPK and PPARγ pathways [12]. Overall, these data including in our study suggest that α-MG could potentially regulate hepatic steatosis in an obesity setting.

ROS are critical factors in the pathogenesis of NAFLD leading to cellular damage and mitochondrial dysfunction. As evident in our study, MGE enhanced the cellular antioxidant enzyme activities including GPx, GRd, SOD, CAT, and GSH (Table 1), which could explain the substantial decline in tROS (Fig. 2b), and mitoROS (Fig. 2c) in the MGE treatment. The reduced ROS levels implied increased cellular protection as supported by decline in TBARS (Fig. 2a), a potential indicator of oxidative cellular damage due to lipid peroxidation. Mangosteen pericarps are rich sources of xanthones bioactive compounds that could have enhanced the endogenous antioxidant defence. Diminished GSH has previously been implicated in the pathogenesis of alcohol liver diseases [18]. In a previous study, Marquez-Valadez et al. found α-MG (10 μM, 25 μM and 50 μM) significantly increased GPx activity in rat brain synaptosomes with 3-NP-induced mitochondrial dysfunction [19]. Fang et al. studied ARPE-19 cells with H_2_O_2_-induced high cellular stress and found α-MG treatment (200 μM) decreased TBARS levels and increased SOD, GPx and GSH activities [20]. Shen et al. reported effects of α-mangosteen in the regulation of antioxidant sensor NRF2 transcription factor on regulation of transcription and redox status in adipocyte [21]. The authors attributed these observations to α-MG regulation of mitogen-activated protein kinases (MAPKs), extracellular signal-regulated kinase (ERK)1/2, JNK and p38 protein expression [20].

Emerging evidence has shown defects in mitochondrial bioenergetics may have a role in the development of liver diseases especially in an obesity setting [18]. Mitochondrial dysfunction can lead to an imbalanced triglyceride accumulation via reduced mitochondrial fatty acids oxidation mechanism. Therefore, reduced ROS and suppressed TBARS in the αMG treatment suggested mitochondrial protection from oxidative damage. In our study, αMG treatment showed enhanced OCR (Fig. 3d), NCCR (mitochondrial complex I and III) and SCCR (mitochondrial complex II and III) (Fig. 3c), which are critical aspects of cell bioenergetics and mitochondrial oxidative phosphorylation relating to breakdown triglycerides for energy metabolism. In our study, improved mitochondrial oxidative phosphorylation is also evident from the enhanced mitochondrial membrane potential in the αMG treatment (Fig. 3b).

On the other hand, loss of mitochondrial membrane potential is strongly associated with apoptosis signalling via release of Ca^2+^, cytochrome c and caspases. As shown in our study, control group showed markedly reduced mitochondrial membrane (Fig. 3b), which corresponded to enhanced release of Ca^2+^ (Fig. 2e), cytochrome c (Fig. 2d), and caspase activity (Fig. 3a) compared to αMG group. Our findings are comparable to those of Moravcova et al., who found 1 mM fatty acid mix (palmitate: oleate acid, 2:1) treatment increased triglyceride, ROS, caspase 3 and reduced mitochondrial membrane potential (Δψm) in primary liver cells, and further recommended in vitro models as suitable tools for studying hepatocellular consequences of steatosis [22]. Increased apoptosis of hepatocytes can lead to rapid cell death and inability of the cells to effectively modulate the lipid metabolism leading to hyperlipidemia. In our study, incorporation of αMG improved mitochondrial membrane potential and suppressed levels of Ca^2+^, cytochrome c, and caspase activity. Thus, the reduced triglyceride accumulation could partly be attributed to suppressed hepatocellular apoptosis via improved mitochondrial membrane potential and overall improved cell functions.

The alternative explanation for the reduced apoptosis levels in the MGE group can be due to the improved cellular antioxidant capacity as evident in the reduced tROS, increased antioxidant enzyme activities (Table 1), and reduced oxidative damage due to lipid peroxidation (Fig. 2a). ROS plays an important role in mediating apoptosis via redox signaling of ASKI signalosome/JNK pathways [9]. In an earlier study, Janhom and Dharmasaroja, suggested the inhibition effect of α-MG against MPP^+^-induced apoptosis in neuroblastoma SH-SY5Y cells may be associated with the reduction of ROS production, modulation of the balance of pro- and anti-apoptotic genes, and suppression of caspase-3 activation [23]. Kwak et al., in a recent study indicated α-MG treatment suppressed cell death via G1 phase arrest and downregulated cell cycle-related proteins (CDK/cyclin) in human oral squamous cell carcinoma [24]. Shan et al. reported α-MG (7 μg/mL) treatment markedly suppressed human gastric adenocarcinoma cells via the constitutive Stat3 protein activation, and Stat3-regulated Bcl-xL and Mcl-1 protein levels [25]. The question that remains in the current study is the potential molecular targets for the α-MG modulation of hepatic triglyceride accumulation and apoptosis, hence future studies are warranted to elucidate the molecular mechanisms by which α-MG modulates hepatic lipid homeostasis via mitochondrial functionality and apoptosis pathways.

**Fig. 4:**
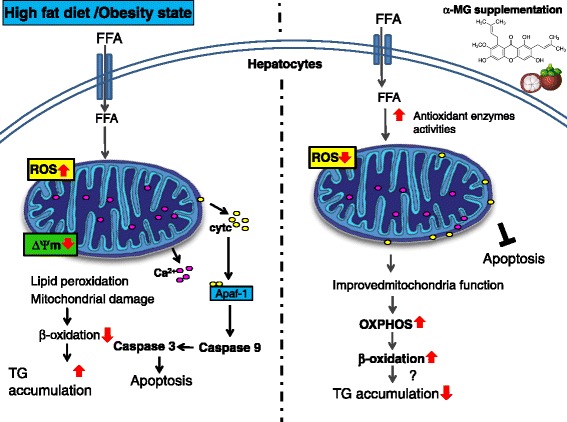
Proposed mechanism for α-MG action against fat infiltration of the liver. High fat diet in an obesity setting, leads to overload of FFAs in hepatocytes. Consequently, there occurs increased ROS production and lipid peroxidation in the liver, which further damage the antioxidant enzymes and mitochondria. Mitochondrial damage leads to cyt *c* and Ca^2+^ release from mitochondria, thereafter cyt *c* binds to Apaf-1 and activates apoptosis via caspases 9 and 3 factors. Apoptosis implies rapid death of hepatocytes hence reduced clearance of fat deposits. On the other hand, mitochondrial damage in turn leads to a decreased mitochondrial oxidative capacity, including β-oxidation and ATP production, resulting in TG accumulation in the liver. α-MG limits hepatic TG accumulation through enhanced anti-oxidative capacity and hepatic mitochondrial oxidative metabolic capacity and inhibition of mitochondrial pathway of apoptosis, which may confer decreased hepatic steatosis risk. ROS: reactive oxygen species; Ca^2+^ : calcium ; cyt *c* : cytochrome *c* ; TG: triglyceride

Overall, Fig. 4 describes our proposed model for the α-MG regulatory effect on hepatic steatosis in high fat diet fed rats. High fat diet led to overload of FFA/triglycerides, increased lipid peroxidation and ROS production in the liver, further suppressing the antioxidant capacity and causing mitochondrial damage. Mitochondrial damage led to increased cytochrome *c* and Ca^2+^ release from mitochondria, and subsequent activation of caspases 9 and 3, eventually leading to hepatocyte apoptosis via mitochondria-dependent pathways. The mitochondrial damage in turn leads to a decrease in mitochondrial oxidative capacity, including β-oxidation and ATP production, and consequently excessive triglyceride accumulation in the liver. Upon supplementation of high fat diet with α-MG from mangosteen pericarps, the antioxidant enzymes activities were enhanced, leading to decreased ROS levels and increased hepatic mitochondrial oxidative phosphorylation capacity and efficient catabolism of accumulated hepatic triglyceride. On the other hand, α-MG resulted in decreased hepatocyte apoptosis via improved mitochondrial integrity and diminished oxidative stress. This means that the liver cells role in lipid metabolism were not dysregulated by the programmed cell death arising from mitochondria-dependent apoptosis. Chitchumroonchokcha et al. studied the bioavailability of xanthones in human subjects and observed all the participants completed the study with no gastrointestinal distress after consuming the test sample of 60 mL mangosteen juice (59% α-mangostin) [26]. The findings of this human study are a good indication on the safety of the α-MG extract and the potential application of the α-MG in therapeutic management of NAFLD.

## Conclusion

Our study demonstrates that α-MG suppressed hepatic steatosis in high fat diet- fed rats via various mechanisms including increased antioxidant enzyme capacity, improved mitochondrial functionality and suppressed mitochondria-dependent apoptosis. These data indicate α-MG extracts from mangosteen pericarps may be used as a preventive agent for hepatosteatosis through regulating mitochondrial oxidative phosphorylation and apoptosis mechanisms.

## Abbreviations

AMPK: Denosine 5’-monophosphate (AMP)-activated protein kinase
Ca^2+^: Calcium
CAT: Catalase
CCCP: Carbonylcyanide p-trifluoromethoxyphenylhydrazone
cyt *c*: cytochrome *c*
ERK: Extracellular signal-regulated kinase
ETC: Electron transport chain
FBS: Fetal bovine serum
FFA: Free fatty acid
GPx: Glutathione peroxidase
GRd: Glutathione reductase
GSH: Glutathione
HFD: High-fat diet
MAPKs: Mitogen-activated protein kinases
MDA: Malondialdehyde
MGE: Mangosteen pericarp extract
mitoROS: mitochondrial ROS
NAFLD: Nonalcoholic fatty liver disease
NCCR: NADH-cytochrome *c* reductase
SCCR: Succinate-cytochrome *c* reductase
SD: Sprague Dawley
SOD: Superoxide dismutase
TBARS: Thiobarbituric acid-reactive substances
TG: Triglycerides
tROS: total reactive oxygen species
α-MG: α- mangostin
Δψm: Mitochondrial membrane potential
